# Nanoformulation Development to Improve the Biopharmaceutical Properties of Fisetin Using Design of Experiment Approach

**DOI:** 10.3390/molecules26103031

**Published:** 2021-05-19

**Authors:** Wan-Yi Liu, Chia-Chen Lin, Yun-Shan Hsieh, Yu-Tse Wu

**Affiliations:** 1 School of Pharmacy, Kaohsiung Medical University, Kaohsiung 80708, Taiwan; sh980713@gmail.com (W.-Y.L.); nn961620@gmail.com (C.-C.L.); u105003038@gap.kmu.edu.tw (Y.-S.H.); 2 Drug Development and Value Creation Research Center, Kaohsiung Medical University, Kaohsiung 80708, Taiwan

**Keywords:** fisetin, nanoparticles, central composite design, intestinal permeability

## Abstract

This study aimed to design an effective nanoparticle-based carrier for the oral delivery of fisetin (FST) with improved biopharmaceutical properties. FST-loaded nanoparticles were prepared with polyvinyl alcohol (PVA) and poly(lactic-co-glycolic acid) (PLGA) by the interfacial deposition method. A central composite design of two independent variables, the concentration of PVA and the amount of PLGA, was applied for the optimization of the preparative parameter. The responses, including average particle size, polydispersity index, encapsulation efficiency, and zeta potential, were assessed. The optimized formulation possessed a mean particle size of 187.9 nm, the polydispersity index of 0.121, encapsulation efficiency of 79.3%, and zeta potential of −29.2 mV. The morphological observation demonstrated a globular shape for particles. Differential scanning calorimetry and powder X-ray diffraction studies confirmed that the encapsulated FST was presented as the amorphous state. The dissolution test indicated a 3.06-fold increase for the accumulating concentrations, and the everted gut sac test showed a 4.9-fold gain for permeability at the duodenum region. In conclusion, the optimized FST-loaded nanoparticle formulation in this work can be developed as an efficient oral delivery system of FST to improve its biopharmaceutic properties.

## 1. Introduction

**Figure 1:**
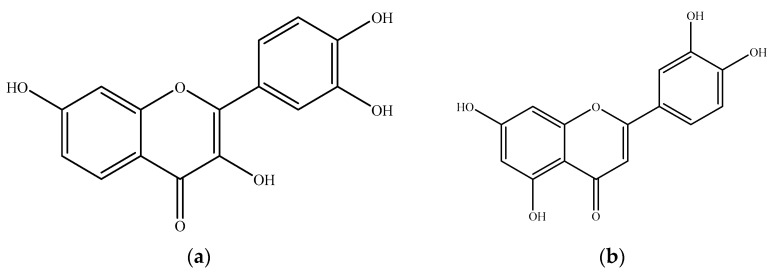
Chemical structures of (**a**) fisetin and (**b**) luteolin (as the internal standard for HPLC analysis).

Fisetin (3,7,3′,4′-tetrahydroxyflavone, FST) (Figure 1) is a naturally occurring flavonol, which is widely distributed in common daily vegetables and fruits, such as strawberry, apple, persimmon, onion, and cucumber. Among them, strawberry processed by lyophilization contains the highest concentrations of FST (160 μg/g) [1,2]. A growing number of studies have also suggested that FST possesses health-promoting potentials to alleviate several oxidative stress-associated diseases, such as metabolic syndrome and neuron degeneration. A high fructose-fed mice model was applied to examine the modulation of FST on metabolic dysfunction, and the results found that an 8-week supplementation of FST (10 mg/kg) resulted in positive effects on insulin resistance, dyslipidemia, and serum inflammatory markers [3]. Since FST could inhibit the peroxidation of 5-lipoxygenase, increase the glutathione level, then reduce the inflammatory response, FST could prevent the incidence of arteriosclerosis and neuron damage [4,5,6,7]. Several in vitro and in vivo studies have reported the neuron-protective effects of FST. Zheng et al. [8] proved that FST could inhibit the activation of microglia, which would produce proinflammatory cytokines and ROS, thereby reduce the neurotoxicity with the BV−2 cell model. Maher et al. [9] utilize a dual in vivo model, *Drosophila* and R6/2 mouse model, to demonstrate that FST has the potential to treat Huntington’s disease through the inhibition of the ERK pathway. A recent study has examined various flavonoid compounds using oxidative and genotoxic stress tests in the senescent murine model, and the results indicated that FST supplementation is significantly beneficial to aged wild-type mice by regaining their tissue homeostasis, reducing the age-related pathology, and extending lifespan, which proves FST has senolytic effect [10]. The health-promoting effects of FST have also attracted the attention of Mayo Clinic; they proposed several FST-targeted clinical trials aimed at the senolytic, anti-COVID 19, and antidiabetic effects [11].

Despite FST’s profound health-promoting effects, the efficient oral delivery of FST is still problematic because the low aqueous solubility (less than 1.0 mg/mL) limits its dissolution and oral bioavailability [12,13]. In addition, the parent form existed transiently and was rapidly biotransformed to sulfates and glucuronides in the rat serum when FST was administered orally, suggesting the extensive first-pass metabolism through the intestine and liver [14,15]. The biopharmaceutical classification system (BCS) classifies active pharmaceutical ingredients into four groups according to solubility and permeability [16], which are key factors for assessing oral bioavailability. In view of therapeutic application, improvement of solubility and permeability is a major approach to enhance oral absorption. In order to improve the biopharmaceutical properties of FST for better absorption, different formulation strategies have been proposed, such as nanoemulsion [17], liposome [13], and nanoparticles [18,19]. The nanoparticle composed of biodegradable polymers is a useful strategy to ameliorate the absorption and therapeutic effects of bioactive components. Among those pharmaceutical polymers, poly(lactic-co-glycolic acid) (PLGA) has attracted the most attention, due to its biocompatible, biodegradable, and low toxic properties, and this material is approved for pharmaceutical uses by the US Food and Drug Administration [20]. Bioactive compounds encapsulated in nanoparticles contribute to the increased solubility, reduced first-pass degradation in the gastrointestinal tract, and also the controlled release of active ingredients [21]. 

For the development of ideal nanoparticle formulation, various preparative parameters, such as the amount of polymer and surfactant, should be taken into consideration, because these parameters affect the resulting particle size, size distribution, encapsulation efficiency, and zeta potential, which are critical factors for the dissolution rate, permeability, and stability. 

The design of experiment (DOE) approach is a method to optimize the preparative variables for the development of FST-loaded PLGA nanoparticles (FST–NP). DOE may provide an alternative to assess the impacts of many factors on responses more efficiently than one-factor-at-a-time (OFAT) experiments [22]. The advantages of DOE include that the interaction between factors can be evaluated comprehensively with fewer runs of experiments and time [23]. Theoretically, smaller particle size, greater surface area, and higher adhesion to the biomembrane are the key factors, which could enhance the oral bioavailability of nanoparticles through better intestinal permeability [20]. Therefore, responses in the study were set as particle size, PDI, encapsulation efficiency, and zeta potential to assure the ideal physicochemical properties. There is only one report about the FST nanoparticle optimized by DOE. Feng et al. [19] employed Taguchi orthogonal array design to optimize the FST–polylactic acid (PLA) nanoparticle, and the responses were set as encapsulation efficiency, particle size, and drug release ratio. However, a higher drug release ratio does not mean higher absorption. To ensure absorption efficiency, the everted gut sac study was adopted to elucidate the permeability of optimized FST–NP in different intestinal sections. This study aimed to develop and optimize the FST–NP for improving the biopharmaceutical characteristics, such as dissolution, permeability, and stability of FST. The response surface methodology (RSM) based on central composite design (CCD) was applied to comprehensively evaluate the impacts of variables on the physicochemical properties of FST–NP, and we assessed the optimized formulation for its biopharmaceutical and physicochemical characteristics.

## 2. Results and Discussion

### 2.1. Determination and Analytical Method Validation of FST

**Figure 2:**
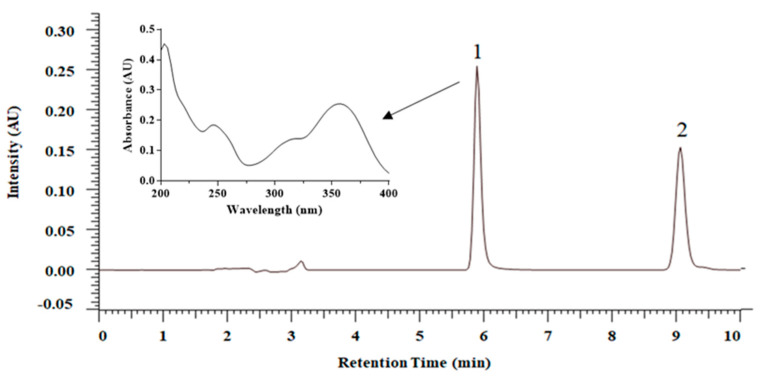
HPLC–DAD chromatogram and UV spectrum of fisetin (25.0 μg/mL) and luteolin (as internal standard) (25.0 μg/mL): 1. fisetin; 2. luteolin.

An HPLC–DAD method was used for quantification of FST in the formulation and in vitro release study, and the analytical chromatogram is shown in Figure 2. The retention time of FST is 5.9 min and the internal standard (luteolin) is 9.1 min. The equation of the calibration curve is y = 0.0497x + 0.0016. The coefficient of determination is 0.9999 for the concentration range from 0.5 to 50 μg/mL. The lower limit of detection is 0.1 μg/mL, and the limit of quantification (LOQ) is 0.5 μg/mL. The accuracy is between 95.3 and 102.2%, and precision is between 0.9 and 3.7%, suggesting that this analytical method has good accuracy and precision.

### 2.2. Formulation Optimization of FST–NP by CCD

The development of nanoformulations with desired properties relies on comprehensive process parameters. We conducted CCD to discuss the effect of PVA concentration and the amount of PLGA. In this experimental design, the total number of experimental groups is 13. Table 1 reveals the responses, which are prepared by different factors and levels. In Table 1, the particle size of FST–NP is 102.9–244.4 nm, EE is 42.8–83.6%, zeta potential is between −7.68 and −25.94 mV, and PDI is 0.053–0.792.

#### 2.2.1. The Effect on Particle Size (Y_1_)

**Figure 3:**
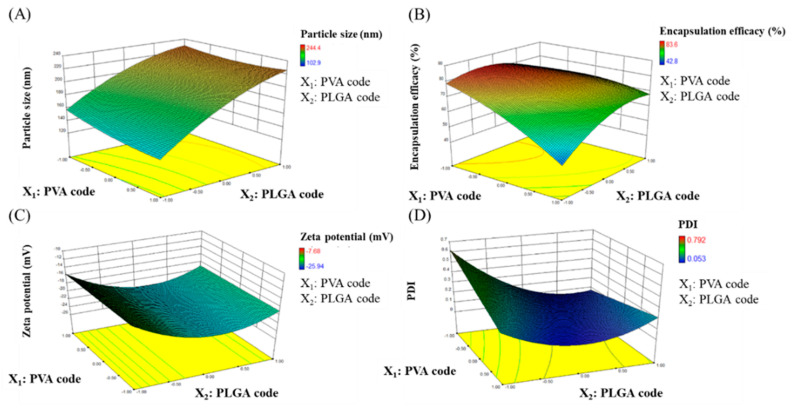
Response surface plots of PVA concentration (X_1_) and amount of PLGA (X_2_) for (**A**) particle size (Y_1_), (**B**) encapsulation efficiency (Y_2_), (**C**) zeta potential (Y_3_), and (**D**) PDI (Y_4_) analysis.

Particle size plays an important role in this study and has a crucial influence on the characterization of FST–NP. In the study, the ANOVA statistical analysis and model fitting were conducted to realize the relationship between factors and responses of FST–NP. In this model, the *p*-value of X_2_ (PLGA amount) and X_2_^2^ are below 0.05, indicating they would have an influence on particle size and concluded to Equation (1). In Figure 3A, the particle size of FST–NP increases substantially as the PLGA amount rises, and PVA concentration would not obviously affect the particle size of FST–NP. In previous studies, Song et al. [24] indicated that the addition of PLGA would increase the viscosity of the organic phase and thus hinder the dispersion of the organic phase into the aqueous phase, resulting in a bigger particle size of the nanoparticles, which is in accordance with our results.

Particle size (Y_1_) = 198.2 + 40.52X_2_ − 13.3X_2_^2^ (1)

#### 2.2.2. The Effect on Encapsulation Efficiency (Y_2_)

EE would display the preparation efficiency of this research. For EE, the *p*-values of X_1_ (PVA concentration), X_2_ (PLGA amount), X_1_X_2_, X_1_^2^, and X_2_^2^ are all less than 0.05, suggesting that these variables have significant differences in the EE response (Equation (2)). In Figure 3B, the encapsulation efficiency may decrease with higher PVA concentration and less PLGA amount. From the results of previous work [24], the solubility of FST in the water phase will increase when PVA concentration increases, bringing about the lower distribution of FST to the organic phase. It leads to a decline in encapsulation efficiency, which is consistent with our study. Feczkó et al. [25] encapsulated bovine serum albumin (BSA) in PLGA nanoparticles with Box–Behnken experimental design, one model of DOE, and also found that EE would positively affect by PLGA concentration. In some studies regarding flavonoids loaded PLGA nanoparticles, EE showed a rising trend when PLGA increased from 5–40 mg/mL because of the elevated viscosity [26]. In our study, PLGA concentration ranges from 1.67 mg/mL to 38.3 mg/mL, which results in a trend similar to previous studies.

Encapsulation efficiency (Y_2_) = 77.68 − 9.05X_1_ + 3.68X_2_ + 7.63X_1_X_2_ − 3.98X_1_^2^ − 7.90X_2_^2^ (2)

#### 2.2.3. The Effect on Zeta Potential (Y_3_)

Zeta potential provides information about the stability for FST–NP dispersion. The higher the absolute value of this response is, the better stability of FST–NP performs because of electrostatic repulsion. For zeta potential, the *p*-value of X_2_^2^ (square of PLGA amount) is below 0.05, indicating FST–NP is significant affected by the amount of PLGA (Equation (3)). In Figure 3C, the zeta potential would decline first and then boost with the addition of PLGA but was not affected by PVA concentration. The negative charge of FST–NP was caused by the existence of terminal carboxylic groups of PLGA. Therefore, the more PLGA added, the more negative the surface charge was. Zeta potential of PLGA nanoparticle without surfactant during preparation is −45 mV owing to dissociation of carboxyl groups on the surface [27], and it would rise when PVA concentration increases from 0.5% to 5% due to surface shading [28]. In our study, PVA concentration is set to smaller, ranging from 0.295% to 1.705%, and therefore, the difference of zeta potential is little.

Zeta potential (Y_3_) = −21.71 − 1.67X_2_ + 4.51X_2_^2^ (3)

#### 2.2.4. The Effect on PDI (Y_4_)

PDI is a measure of homogeneity of particle size and is generally expressed as smaller than 0.3 for a homogenous population of nanocarriers [29]. After ANOVA analysis, the *p*-values of variables X_2_ (PLGA amount), X_1_X_2_, and X_2_^2^ are less than 0.05, which means that these variables have significant effects on PDI (Equation 4). In Figure 3D, PLGA amount does not have an influence on size distribution. However, low PVA concentration may obviously increase PDI value and make formulation uneven, leading to lower efficiency of drug delivery.

PDI (Y_4_) = 0.1 − 0.056X_1_ − 0.15X_2_ + 0.13X_1_X_2_ + 0.19X_2_^2^ (4)

The results show that the optimal formulation contains a PVA concentration of 0.5% and a PLGA amount of 75.3 mg. According to this formulation, PLGA nanoparticles were prepared, and the particle size, encapsulation efficiency, zeta potential, and PDI were displayed in Table 2. After the experiment, the particle size and EE of optimized FST–NP are fitted to values predicted by DOE with an error below 5%. The value of zeta potential and PDI are lower than the predicted one, which is beneficial for better stability and particle distribution. Kadari et al. [30] encapsulated the FST–cyclodextrin inclusion complex into PLGA nanoparticles. The particle diameter was 87.27 ± 0.10 nm, the zeta potential was −8.71 ± 0.03 mV, and EE was 79%. The EE was decreased substantially to 47.3%, without the addition of cyclodextrin. In our study, we proposed a straightforward method, which applied merely PLGA to yield FST–NP with better EE and ideal zeta potential (<−20 mV). 

After optimization, it seems that a particle size of 187.9 nm, with a PDI of 0.121, is comparable [19,31]. As listed in Table 3, Ghosh et al. [31] employed human serum albumin (HSA) to develop FST–HSA nanoparticles, possessing a particle size of 220 nm, and encapsulation efficiency (EE) of 84%. Feng et al. [19] optimized FST–PLA nanoparticles with Taguchi orthogonal array design, and the particle size was relatively bigger due to higher EE. Zeta potential is the key indicator for nanoparticle stability. If the absolute value of zeta potential is lower than 20, it would be less stable because of the possibility of flocculation. FST–PLA [19] and HPβ-CD/PLGA [30] nanoparticles have the zeta potential of −15.63 mV and −8.71 mV, respectively, which might lead to shorter storage periods. The FST–PLGA nanoparticle has a zeta potential of −29.2 mV, which is beneficial for long-term storage stability. 

### 2.3. Characterization of FST–NP

#### 2.3.1. Morphological Study of FST–NP

**Figure 4:**
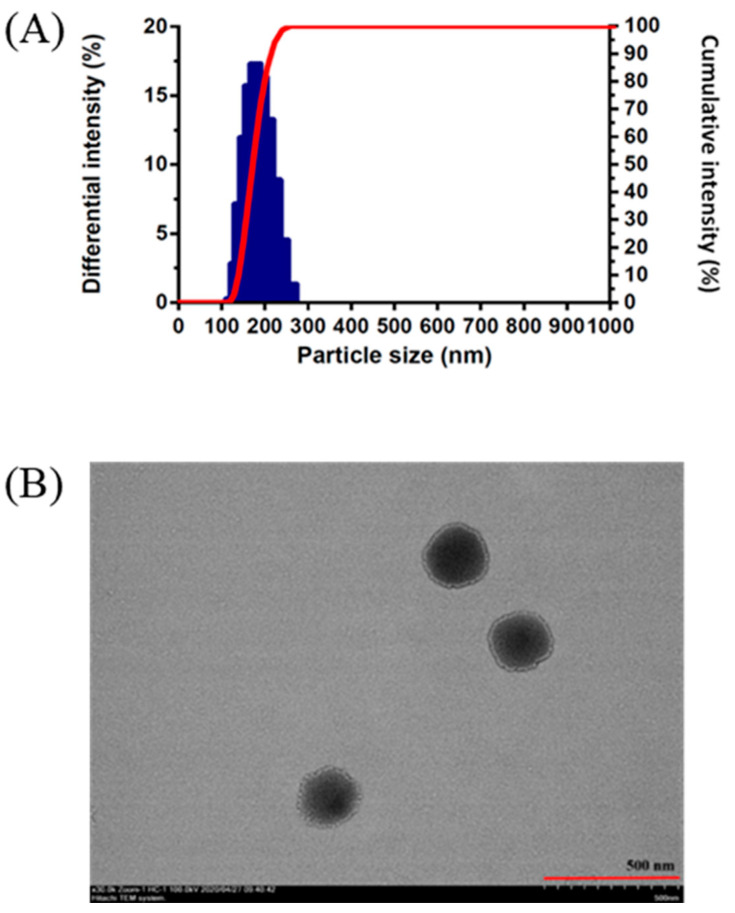
The particle size measured by (**A**) DLS and (**B**) transmission electron microscopy image of FST–NP optimized formulation.

The particle size measured by DLS is 187.9 ± 6.1 nm (Figure 4A). The particle size is superior to that of previous studies, where the particle size is about 220 nm [19,31]. Moreover, the FST–NP displayed spherical shape and even distribution in Figure 4B, which are consistent with previous studies [19,30].

#### 2.3.2. Differential Scanning Calorimetry

**Figure 5:**
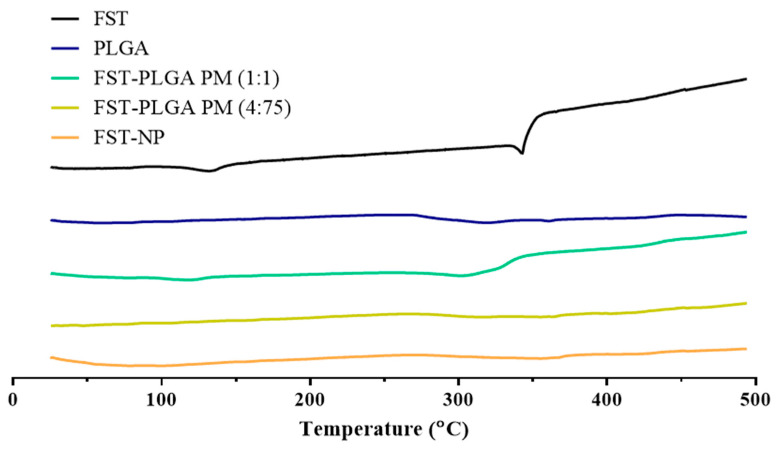
DSC thermogram of FST, blank PLGA nanoparticles, physical mixture 1:1, physical mixture 4:75, and FST–NP.

For characterization of FST–NP, the differential scanning calorimetry was executed to confirm its thermal performance. The endothermic peaks of the FST appeared at 132 °C and 343 °C for water loss and the melting point of FST, respectively [32]. For the physical mixture, two ratios were prepared to present the thermal properties of FST and PLGA completely and meet the actual condition of FST–NP. In Figure 5, the blank group PLGA nanoparticles, the physical mixing group, and FST–NP have no obvious endothermic peaks, suggesting that FST–NP may be the amorphous state and encapsulated in PLGA nanoparticle.

#### 2.3.3. Powder X-ray Diffraction

**Figure 6:**
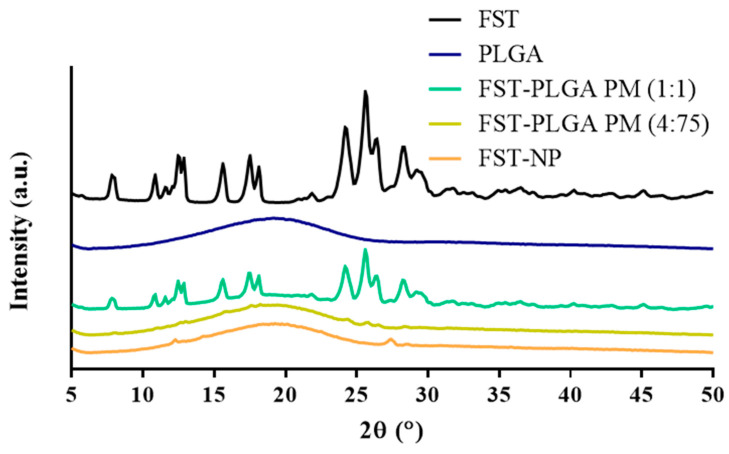
PXRD pattern of FST, blank PLGA nanoparticles, physical mixture 1:1, physical mixture 4:75, and FST–NP.

Powder X-ray diffraction was used to affirm the crystallinity of FST–NP. In Figure 6, the diffraction peaks (2θ) of FST appeared at 7.91°, 10.85°, 12.55°, 12.84°, 15.63°, 17.49°, 18.14°, 24.25°, 25.65°, 26.37° and 28.30°, suggesting that FST is present as a highly crystalline structure [33,34]. There is no observed diffraction peak of the blank group PLGA, indicating PLGA is present as an amorphous state. In the physically mixed group, the intensity of the diffraction peak is weak in the FST–PLGA PM (4:75), while mixing at 1:1(*w*/*w*) ratio group have obvious diffraction peaks owing to different dilute ratios. For FST–NP, there are no peaks observed in the PXRD pattern, speculating that FST may be evenly dispersed in PLGA and become amorphous after preparation.

#### 2.3.4. Fourier Transform–Infrared Spectroscopy

**Figure 7:**
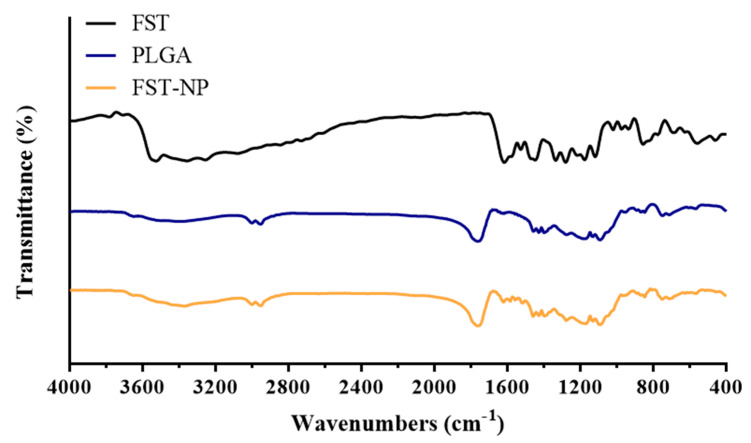
FT–IR spectroscopy of FST, blank PLGA nanoparticles, and FST–NP.

FT–IR was used to confirm that FST was encapsulated in PLGA nanoparticles. In Figure 7, the characteristic peak positions of FST appeared at 3522 cm^−1^ (OH stretching), 3356 cm^−1^ (OH stretching), 1615 cm^−1^ (C=O stretching), 1447 cm^−1^ (aromatic C=C stretching), 1280 cm^−1^ (–OH bending), 1120 cm^−1^ (C–O stretching) and 854 cm^−1^ (out of plane C–H bending) [31,34,35]. The characteristic peak positions of the PLGA nanoparticles in the blank group appeared at 2998 cm^−1^ (C–H stretching), 2950 cm^−1^ (C–H stretching), and 1760 cm^−1^ (C=O stretching) [36]. In the FST–NP group, the observable PLGA peak positions at 2998 cm^−1^, 2950 cm^−1^, and 1760 cm^−1^ did not change and was similar to that of blank PLGA nanoparticle because of the relatively low content of FST. Compared with FST, the characteristic peaks showed displacement from 3356 cm^−1^ to 3370 cm^−1^, and from 1615 cm^−1^ to 1622 cm^−1^. It showed that FST is present in the matrix of PLGA, and there may be some interactions among them [37].

### 2.4. In Vitro Release

**Figure 8:**
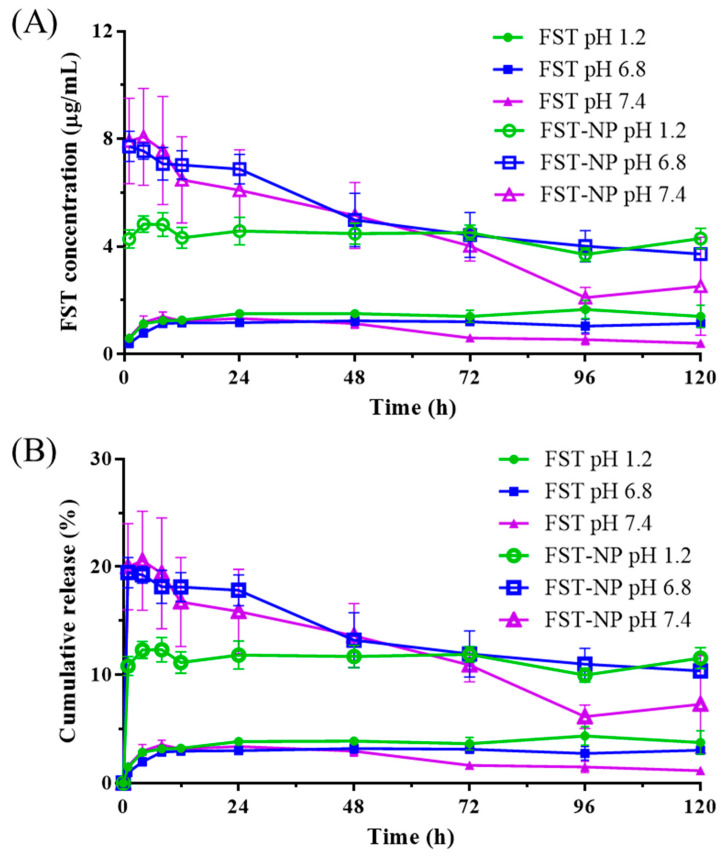
The in vitro release profile of FST and FST–NP in the pH 1.2, 6.8, and 7.4 medium by presenting (**A**) concentration and (**B**) percentage of cumulative release. Data are expressed as mean ± standard deviation (*n* = 3). Green solid and hollow circle present FST and FST–NP in pH 1.2, blue solid and hollow square present FST and FST–NP in pH 6.8, and purple solid and hollow triangle present FST and FST–NP in pH 7.4.

In the pH 1.2, pH 6.8, and pH 7.4 release medium, the release of FST–NP are all higher than the FST standard, and the release reaches a stable level after the first hour (Figure 8). In this study, the release of FST within 24 h is between 10.8 and 19.9%, and the highest release is presented in the pH 7.4 medium, which fits the solubility in different pH conditions. In the pH 1.2 medium, the concentration of FST did not decrease significantly after 120 h; however, FST concentration is observed to decline in the alkaline condition. Despite higher solubility in the alkaline environment [38], the pKa of the OH group at the C7 position of FST is 7.27 in an aqueous and neutral medium. Therefore, when the pH is adjusted to 7.4, it would cause the deprotonation of FST, as shown in Figure S1 [39,40]. In this study, the release of FST within 24 h is between 10.8 and 19.9%, and the highest release is presented in the pH 7.4 medium, which fits the solubility in different pH conditions. For the mechanism of drug release, it displays biphasic with burst release and sustained release section. Burst release would occur because of interfacial deposition at the first 24 h, while PLGA matrix erosion takes place after 24 h, and sustained zero-order model release was observed [41].

### 2.5. Permeability Evaluation of FST–NP

**Figure 9:**
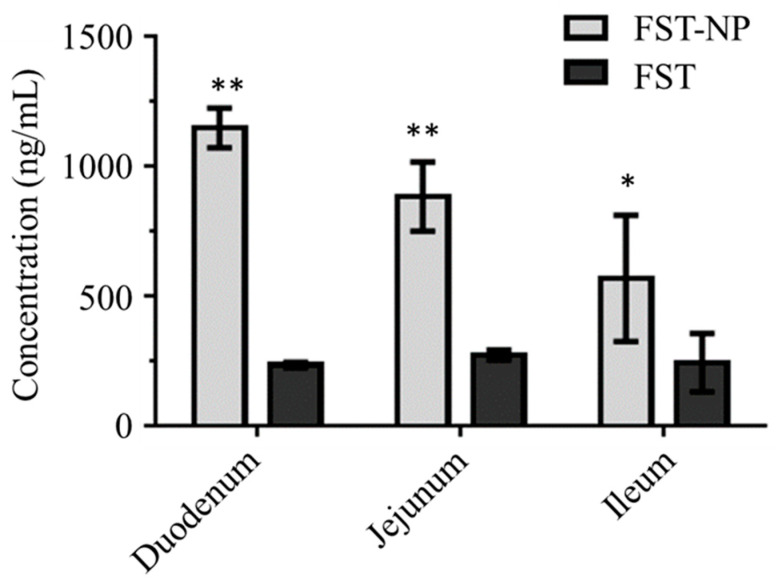
The permeability evaluation of FST and FST–NP. Data are expressed as mean ± standard deviation. (*n* = 3); *, ** indicated that FST–NP is significantly different compared with the FST group (*p* < 0.05).

In Figure 9, the optimized FST–NP could significantly enhance intestinal permeation in the whole intestine, especially in the duodenum and jejunum. In this test, the amount of FST standard that penetrated into the duodenum, jejunum, and ileum are 233, 272, and 243 ng/mL, respectively. After preparation, the permeation of FST would be 4.9, 3.2, and 2.3 times higher than suspension, suggesting FST–NP may significantly enhance intestinal permeation. Ligated ileum loop model was conducted to evaluate intestine absorption of PLGA nanoparticles previously, and it found that the fluorescence intensity of duodenum was strongest because of higher concentration and became weaker gradually in the posterior intestine. A previous study [42] found that the drug loaded in PLGA nanoparticles had a higher uptake than the prototype, and the results are in accordance with ours. There are some Peyer’s patches or other specific tissues in the intestines of humans and animals, which account for about 25% of the whole intestinal mucosa, and it is the main site of the uptake of nanoparticles. Due to its smaller size, higher surface area, and better adhesion to the biofilm, nanoparticles will accumulate and enter the intestine [43].

### 2.6. Storage and pH Stabilities of FST–NP

In this study, the stability test of FST–NP was conducted at 5 ± 3 °C and 25 ± 2 °C, 60 ± 5% RH and observed the particle size, PDI, and FST content of FST–NP after 0, 3, 7, 14, 21, 30 and 60 days. Table 4 shows that the particle size does not change significantly after a 60-day storage period, and the PDI value is also within 0.3, indicating that the nanoparticles do not agglomerate during storage. Ghosh et al. [31] conducted a three-month storage study to evaluate the stability of FST-loaded human serum albumin nanoparticles, and the particle size, PDI, and morphology did not change significantly. The result is consistent with ours. In addition, the FST content is not lower than 90% of the original content after storage, indicating that the preparation of PLGA nanoparticles can protect the FST from degradation. Therefore, the results of FST–NP suggest that PLGA nanoparticles are able to improve the stability of FST and prevent it from degradation.

In a previous study, FST was demonstrated to have a concerning level of stability in alkaline conditions, and the half-life of FST in pH 7.5 condition at 37 °C is only 3.4 h [39]. This property would affect the therapeutic efficiency of FST. Hence, we conducted a pH stability study to evaluate FST stability at different pH conditions. In Table 5, the concentration of FST in pH 1.2 does not change significantly after 72 h. Yet, in pH 6.8 and 7.4 conditions, FST concentration is decreased to 1.72 μg/mL and lower than LOQ, and the half-life is 46.6 and 2.8 h after calculation, respectively. This result shows that FST has worse stability and a faster degradation rate in alkaline conditions, which agrees with the previous literature [39]. However, the half-life of FST–NP is about 96 h at pH 6.8 and about 72 h at pH 7.4, suggesting PLGA can prevent FST from degradation in the alkaline conditions.

The utilization of flavonoids as therapeutic or health-promoting agents is generally hindered by their inherent properties, such as inferior water solubility, low intestinal permeability, and insufficient stability, which limits the oral absorption of flavonoids. The enhancing of saturation solubility and/or membrane permeability is an effective strategy necessary to improve the oral bioavailability of flavonoids according to the theory of the biopharmaceutical classification system [44]. There are several studies related to the formulation design of encapsulating flavonoids in PLGA nanoparticles. Quercetin-loaded PLGA nanoparticles achieved higher cellular uptake than that of quercetin because of the smaller size for enhancing the permeability and retention (EPR) effect [45]. In addition, Ding et al. [7] designed luteolin-loaded Her-2-PLGA nanoparticles and observed that nanoformulation had a better therapeutic effect on the gastric cancer cells. Furthermore, kaempferol for cancer treatment had also been formulated into PLGA nanoparticles, which showed selective inhibition effect on cancer cells and less harmful effects to normal cells in ovarian [46]. The PLGA nanoparticle formulation could be a promising strategy for cancer treatment due to its improved ability to reduce cancer cell viability. The results suggest that flavonoids-loaded PLGA nanoparticles have great potentialities for clinical application. The PLGA nanoparticle formulation has been developed for the oral delivery of epirubicin [47]. Compared to the epirubicin solution, a 4.49-fold higher permeation of epirubicin across the rat ileum was achieved by this nanoformulation, which also contributed to a 3.9-fold improvement of relative oral bioavailability. In addition, the PLGA nanoformulation was applied to improve the oral absorption of tenofovir disoproxil fumarate [48]. The everted gut sac study proved that using PLGA nanoparticles as a carrier significantly increased the transport flux by fivefold, and the in vivo pharmacokinetic study also demonstrated this formulation efficiently improve the relative oral bioavailability by 6.79-fold when compared with the unformulated drug.

Based on the results above, FST–NP enhances dissolution performance and intestinal permeation via nanoscale size, higher surface area, and better biocompatibility to prolong FST–NP retention time on the intestine. In addition, FST–NP is stable within 60 days to prevent degradation. In this way, FST–NP may enhance oral bioavailability because of characterization improvement and be a potential agent to make therapy efficient.

## 3. Materials and Methods

### 3.1. Materials

Fisetin (purity 96%) was purchased from Alfa Aesar (Haverhill, MA, USA). Luteolin (purity 98%) was from Tokyo Chemical Industries (Tokyo, Japan). PLGA (50:50, Mw 7000–17,000, Resomer^®^ RG 502H) was purchased from Evonik Industries AG (Essen, Germany). Poly (vinyl alcohol) (Mw 9000–10,000) and sodium phosphate monobasic were purchased from Sigma-Aldrich Co. (St. Louis, MO, USA). Acetone was purchased from Echo Chemical Co. (Miaoli County, Taiwan). HPLC grade acetonitrile was obtained from Fisher Scientific (Hampton, NJ, USA).

### 3.2. HPLC–DAD Analysis of Fisetin

In vitro FST content was determined by high-performance liquid chromatography (HPLC), coupled with a diode array detector (DAD) modified from the previous study [49]. The HPLC system (Hitachi 7000, Hitachi, Tokyo, Japan) consisted of a D−7100 pump, an L−7200 autosampler, and an L−7455 DAD detector. FST was separated by a reversed-phase column (Synergi fusion RP, 250 × 4.6 mm, 4 µm; Phenomenex, Inc., Torrance, CA, USA). The determination was conducted at room temperature (27 ± 2 °C). The mobile phase consisted of acetonitrile 0.01 M NaH_2_PO_4_ (pH 2.5) with a ratio of 35:65 (*v*/*v*), and the flow rate was set to 1.2 mL/min. The injection volume was 20 μL and detected at 358 nm. The run time of FST analysis was 10 min. Analytical method validation for FST analysis was performed according to our previous work [50].

For the standard preparation, FST (5.0 mg) standard was dissolved in 50% (*v*/*v*) methanol as stock solution, and diluted to the concentration of 50.0, 25.0, 12.5, 5.0, 1.25, and 0.5 μg/mL with 50% (*v*/*v*) methanol. After dilution, A 25 μL luteolin standard (100.0 μg/mL) was added as internal standard for the preparation of calibration curves.

### 3.3. HPLC–Electrochemical Analysis of Fisetin

The determination of FST in the permeability study was determined by HPLC coupled with an LC–4C amperometric detector (BAS, West Lafayette, IN, USA). The HPLC system (Hitachi 5000, Hitachi, Tokyo, Japan) consisted of a D-5160 pump, an L-5260 autosampler, and a BAS LC–4C electrochemical detector (BAS, West Lafayette, IN, USA). For separation, the same column, flow rate, and injection volume were used as mentioned above. The mobile phase consisted of acetonitrile and 0.05 M NaH_2_PO_4_ (pH 2.5) with a ratio of 23:77 (*v*/*v*). For detection, Ag/AgCl was chosen to be the reference electrode, applied potential was set to +1.0 V, the filter was 0.1 Hz, and the range was 20 nA. 

### 3.4. Preparation of FST–NP 

The FST–NP was prepared by the nanoprecipitation method [26]. PLGA and FST were dissolved uniformly in acetone as organic phase, and PVA solution was prepared as the aqueous phase. The organic phase was added to the aqueous phase at a rate of 0.5 mL/min, and the aqueous phase was maintained for the magnetic stirring of 600 rpm to form a suspension of FST–NP. Acetone was removed by rotary evaporator and stirred overnight to completely evaporate the solvent. Finally, the nanoparticle suspension was centrifuged at 2,5000× *g* for 30 min, the supernatant was removed and FST–NP was collected. 

### 3.5. Formulation Optimization and Characterization of FST–NP

The Design-Expert 6.0.3 software (StatEase Inc., Minneapolis, MN, USA) was applied to conduct RSM based on CCD experiments to optimize the FST–NP formulations. The chosen factors were the concentration of PVA (X_1_) and PLGA amount (X_2_), and responses included particle size (Y_1_), size distribution expressed by the polydispersity index (Y_2_), encapsulation efficiency (Y_3_), and zeta potential (Y_4_), as shown in Table 6. The goals for exploring the optimized FST–NP formulation included the minimized particle size (Y_1_), maximized encapsulation efficiency (Y_2_), zeta potential (Y_3_) < −20 mV and minimized polydispersity index (Y_4_). 

### 3.6. Characterization of FST–NP

#### 3.6.1. Particle Size, Polydispersity Index, and Zeta Potential

FST–NP was diluted 100 times by deionized water [51], and particles size, distribution and zeta potential were measured by dynamic laser scattering (DLS) (ELSZ-2000 particle size analyzer, Otsuka Electronics, Otsuka, Japan). The particle distribution would display by polydispersity index (PDI).

#### 3.6.2. Encapsulation Efficiency (EE)

The EE of FST–NP was evaluated based on previous work [52]. Briefly, the unencapsulated FST was removed by high-speed centrifugation, and FST–NP at the bottom of the centrifuge tube was collected, redissolved in deionized water, and quantified into 10 mL. FST–NP was dissolved in acetonitrile, centrifuged at 9600× *g* for 10 min, and the supernatant was analyzed to evaluate the loaded amount of FST in PLGA nanoparticles and calculated the encapsulation efficiency (EE) with the following equation:

$$\mathrm{EE}\left(\%\right)=\frac{\mathrm{Weight}\;\mathrm{of}\;\mathrm{FST}\;\mathrm{loaded}\;\mathrm{in}\;\mathrm{formulation}}{\mathrm{Weight}\;\mathrm{of}\;\mathrm{FST}\;\mathrm{added}\;\mathrm{for}\;\mathrm{preparation}}\times 100\%$$

### 3.7. Morphological Study of FST–NP

Appearance, shape, and particle size of the FST–NP were observed by transmission electron microscope (TEM) (HT7700, Hitachi, Tokyo, Japan) [53]. FST–NP was diluted with deionized water to an appropriate concentration and dropped onto the carbon-plated copper mesh. After drying, the carbon-plated copper mesh was soaked in 2% (*w*/*v*) phosphotungstic acid aqueous solution, then removed excess phosphotungstic acid, dried, and measurements were determined by TEM.

#### 3.7.1. Differential Scanning Calorimetry

Differential scanning calorimetry (DSC) (Model 404 F3, Netzsch, Bavaria, Germany) was executed to observe melting point and glass transition temperature to evaluate the thermal behavior of FST–NP. In this study, DSC was applied to evaluate the FST, blank PLGA nanoparticles, physical mixture, and FST–NP. The 10 mg sample was weighed in the aluminum pan and heat from 30 °C to 500 °C with a heating rate of 10 °C/min.

#### 3.7.2. Powder X-ray Diffraction

Powder X-ray diffraction (PXRD) (Siemens D5000, Munich, Germany) could be executed to evaluate the change of the crystal form of the FST after preparation. In this study, the PXRD was applied to evaluate the FST, blank PLGA nanoparticles, physical mixture, and FST–NP. The sample was ground before analysis, and the diffraction angle was between 5° and 50° at the scan rate of 0.1°/sec.

#### 3.7.3. Fourier Transform–Infrared Spectroscopy

In this study, Fourier transform–infrared spectroscopy (FT–IR) (Bruker Optics Alpha OPUS, Bruker Corporation, MA, USA) was executed to observe the functional groups and bonding of the FST, blank PLGA nanoparticles, and FST–NP. Sample powder mixed with potassium bromide was compressed into thin tablets for determination. The range of wavenumber was from 4000 to 400 cm^−1^.

### 3.8. In Vitro Release of FST–NP

The in vitro dissolution study was performed by the paddle method (SR8 PLUS dissolution test station, Hanson Research, Chatsworth, CA, USA) to assess the release of the FST–NP in pH 1.2, pH 6.8, and pH 7.4 medium. The sample equal to 4.0 mg of FST would be added to a 100 mL dissolution medium. The temperature was maintained at 37 ± 0.5 °C with a stirring speed of 100 rpm. A total of 500 μL of dissolution medium was collected and refilled 500 μL fresh medium at 1, 4, 8, 12, 24, 48, 72, 96, and 120 h. The withdrawn samples were centrifuged at 13,000× *g* for 10 min and determined by HPLC–DAD (Section 3.2). The results were conducted in triplicate.

### 3.9. Permeability Evaluation of FST–NP

The everted gut sac model [53,54] was executed to know the intestine permeation of FST. The duodenum, jejunum, and ileum were separated, everted, and filled with a tyrode solution. The gut sac was soaked in FST–NP and FST suspension with a concentration of 50 µg/mL and maintained at 37 °C. After 1 h, the solution in the gut sac was collected and analyzed by HPLC–EC (Section 3.3).

### 3.10. Storage and pH Stabilities of FST–NP

The samples were dispensed into sample vials, sealed with rubber stoppers and aluminum caps, and kept at 25 ± 2 °C, 60 ± 5% relative humidity (RH), and 5 ± 3 °C, respectively. The particle size, distribution, and FST content were measured at 0, 3, 7, 14, 21, 30, and 60 days.

Other stability tests for FST were conducted in different pH conditions. FST standard was put in pH 1.2, 6.8, and 7.4 medium with the final concentration of 5 μg/mL for 72 h, and the temperature was maintained at 37 ± 0.5 °C. The sample was withdrawn at 1, 4, 12, 24, 48, and 72 h, and determined by HPLC–DAD.

### 3.11. Data Analysis

The experiment results are expressed as mean ± standard deviation. Differences between formulations used SPSS v20 (SPSS Inc., Chicago, IL, USA) and were compared by *t*-test; *p* < 0.05 was considered a significant statistical difference.

## 4. Conclusions

This study proposed an efficient nanoparticle-based system to improve the biopharmaceutical properties of FST for oral delivery. A straightforward interfacial deposition method was employed to prepare the FST–NP, and the preparative parameters were optimized by the RSM based on CCD to achieve appropriate characteristics, including particle size, PDI, EE, and zeta potential. The optimized FST–NP formulation consists of 5 mg FST, 0.5% (*w*/*v*) PVA, and 75.3 mg of PLGA, resulting in particles with an average size of 187.9 nm, PDI of 0.121, negative surface charge −29.2 mV, and EE of 79.3%. Morphological examination by TEM illustrates globular-shaped nanoparticles. According to the DSC and XRD analysis, FST is enclosed in the PLGA particles as an amorphous state. The FST–NP demonstrates a 3.06-fold increase in the dissolution rate and a 4.9-fold increase in the permeability at the duodenum region. The above results indicate that the FST–NP has great potential to be developed as an oral carrier for efficiently delivering FST with improved biopharmaceutical properties. 

## Figures and Tables

**Table 1:** Experimental design and response of FST–NP.

Run Order	Factors and Levels		Responses			
	X_1_: PVA (%)	X_2_: PLGA (mg)	Y_1_: Particle Size (nm)	Y_2_: EE ^1^ (%)	Y_3_: Zeta Potential (mV)	Y_4_: PDI ^2^
1	0.295	100	224.0	83.6	−20.62	0.152
2	0.5	35	158.3	76.0	−23.51	0.568
3	0.5	165	211.1	71.6	−22.70	0.144
4	1	100	186.4	81.5	−20.52	0.152
5	1	191.5	244.4	65.6	−14.15	0.201
6	1	100	190.3	77.7	−17.34	0.090
7	1	100	198.1	74.2	−22.65	0.053
8	1	100	205.0	77.8	−21.48	0.145
9	1	100	194.1	77.2	−19.86	0.083
10	1	8.35	102.9	60.1	−7.68	0.792
11	1.5	35	145.5	42.8	−12.32	0.144
12	1.5	165	216.8	68.9	−17.36	0.228
13	1.705	100	193.8	57.8	−25.94	0.078

^1^ EE: encapsulation efficiency; ^2^ PDI: polydispersity index.

**Table 2:** Optimal conditions predicted and experimental values of responses.

Factor		Optimal Conditions	
PVA Concentration (%)		0.5	
PLGA Amount (mg)		75.3	
Responses	Predicted Values	Experimental Values	Error (%)
Particle Size (nm)	180.9	187.9 ± 6.1	3.9
EE (%) ^1^	83.1	79.3 ± 2.7	−4.6
Zeta Potential (mV)	−20.3	−29.2 ± 1.6	−43.8
PDI ^2^	0.289	0.121 ± 0.01	−58.1

^1^ EE: encapsulation efficiency; ^2^ PDI: polydispersity index. Data are presented as mean ± standard deviation for each group (*n* = 3).

**Table 3:** Physicochemical characteristic comparison with other FST nanoparticles.

Nanomaterial Type	Particle Size (nm)	PDI	Zeta-Potential (mV)	EE (%) ^1^	Reference
HSA ^2^	220	-	-	84	[31]
PLA ^3^	226.85	0.12	−15.63	90.35	[19]
HPβ-CD/PLGA ^4^	87.27	0.25	−8.71	78.8	[30]
PLGA ^5^	187.9	0.121	−29.2	79.3	This study

^1^ EE: encapsulation efficiency; ^2^ HSA: human serum albumin; ^3^ PLA: poly lactic acid; ^4^ HPβ-CD: hydroxyl propyl beta-cyclodextrin; ^5^ PLGA: poly-lactide-co-glycolic acid.

**Table 4:** Storage stability test of optimized FST–NP in 5 ± 3 °C and 25 ± 2 °C.

Day	5 ± 3 °C			25 ± 2 °C		
	FST Content (%)	Particle Size (nm)	PDI	FST Content (%)	Particle Size (nm)	PDI
0	100.0 ± 1.1	179.3 ± 17.1	0.116 ± 0.016	100.0 ± 1.1	179.3 ± 17.1	0.116 ± 0.016
3	99.1 ± 2.9	178.2 ± 15.4	0.103 ± 0.030	102.1 ± 4.4	176.8 ± 14.2	0.109 ± 0.054
7	96.7 ± 2.9	179.7 ± 15.7	0.085 ± 0.037	100.2 ± 5.0	179.1 ± 12.9	0.093 ± 0.027
14	101.9 ± 0.9	179.3 ± 15.7	0.129 ± 0.037	95.4 ± 3.7	181.7 ± 9.0	0.094 ± 0.013
21	101.6 ± 3.4	179.5 ± 15.9	0.097 ± 0.010	98.2 ± 2.1	180.5 ± 11.6	0.117 ± 0.049
30	99.7 ± 1.0	188.3 ± 22.3	0.108 ± 0.032	103.4 ± 1.7	185.4 ± 7.1	0.138 ± 0.019
60	100.1 ± 3.3	174.9 ± 16.0	0.126 ± 0.043	103.9 ± 2.7	180.7 ± 7.1	0.112 ± 0.021

Data are expressed as mean ± standard deviation. (*n* = 3).

**Table 5:** Degradation assessment of FST in different conditions.

	C_72 h_^1^ (μg/mL)	K ^2^ (h^−1^)	t_1/2_^3^ of FST (h)	t_1/2_ of FST–NP (h)
pH 1.2	4.73	0.001	471.8	N.A. ^4^
pH 6.8	1.72	0.015	46.6	96
pH 7.4	N.D. ^5^	0.245	2.8	72

Initial concentration of FST: 5.0 μg/mL; ^1^ C_72 h_: FST concentration after 72 h; ^2^ K: constant of degradation rate; ^3^ t_1/2_: half-life; ^4^ N.A.: not applicable; ^5^ N.D.: not detectable (sample concentration below limit of quantification).

**Table 6:** Applied dependent factors in the central composite design (CCD).

Independent Variables	Level				
	−1.414	−1	0	1	1.414
X_1_: PVA Concentration (%)	0.295	0.5	1	1.5	1.705
X_2_: PLGA Amount (mg)	8.35	35	100	165	191.5
Dependent Variables	Goal				
Y_1_: Particle Size (nm)	Minimize				
Y_2_: Encapsulation Efficiency (%)	Maximize				
Y_3_: Zeta Potential (mV)	<−20				
Y_4_: Polydispersity Index	Minimize				

## Data Availability

The data presented in this study are available in Supplementary Material.

## Supplementary Materials

The following are available online. Figure S1 HPLC chromatography of FST-NP in pH 7.4 medium at different time points.

## Sample Availability

Samples of the compounds are not available from the authors.

## References

[1] Kimira M., Arai Y., Shimoi K., Watanabe S. (1998). Japanese Intake of Flavonoids and Isoflavonoids from Foods. J. Epidemiol..

[2] Kashyap D., Sharma A., Sak K., Tuli H.S., Buttar H.S., Bishayee A. (2018). Fisetin: A bioactive phytochemical with potential for cancer prevention and pharmacotherapy. Life Sci..

[3] Shi Y.-S., Li C.-B., Li X.-Y., Wu J., Li Y., Fu X., Zhang Y., Hu W.-Z. (2018). Fisetin Attenuates Metabolic Dysfunction in Mice Challenged with a High-Fructose Diet. J. Agric. Food Chem..

[4] Grynkiewicz G., Demchuk O.M. (2019). New perspectives for fisetin. Front. Chem..

[5] Krasieva T.B., Ehren J., O’Sullivan T., Tromberg B.J., Maher P. (2015). Cell and brain tissue imaging of the flavonoid fisetin using label-free two-photon microscopy. Neurochem. Int..

[6] Khan N., Syed D.N., Ahmad N., Mukhtar H. (2013). Fisetin: A dietary antioxidant for health promotion. Antioxid. redox Signal..

[7] Ding J., Li Q., He S., Xie J., Liang X., Wu T., Li D. (2020). Luteolin-loading of Her-2-poly (lactic-co-glycolic acid) nanoparticles and proliferative inhibition of gastric cancer cells via targeted regulation of forkhead box protein O1. J. Cancer Res. Ther..

[8] Zheng L.T., Ock J., Kwon B.-M., Suk K. (2008). Suppressive effects of flavonoid fisetin on lipopolysaccharide-induced microglial activation and neurotoxicity. Int. Immunopharmacol..

[9] Maher P., Dargusch R., Bodai L., Gerard P.E., Purcell J.M., Marsh J.L. (2011). ERK activation by the polyphenols fisetin and resveratrol provides neuroprotection in multiple models of Huntington′s disease. Hum. Mol. Genet..

[10] Yousefzadeh M.J., Zhu Y., McGowan S.J., Angelini L., Fuhrmann-Stroissnigg H., Xu M., Ling Y.Y., Melos K.I., Pirtskhalava T., Inman C.L. (2018). Fisetin is a senotherapeutic that extends health and lifespan. EBioMedicine.

[11] ClinicalTrials.gov. https://clinicaltrials.gov/ct2/results?term=fisetin&Search=Search.

[12] Guzzo M.R., Uemi M., Donate P.M., Nikolaou S., Machado A.E.H., Okano L.T. (2006). Study of the Complexation of Fisetin with Cyclodextrins. J. Phys. Chem. A..

[13] Mignet N., Seguin J., Ramos Romano M., Brullé L., Touil Y.S., Scherman D., Bessodes M., Chabot G.G. (2012). Development of a liposomal formulation of the natural flavonoid fisetin. Int. J. Pharm..

[14] Shia C.-S., Tsai S.-Y., Kuo S.-C., Hou Y.-C., Chao P.-D.L. (2009). Metabolism and Pharmacokinetics of 3,3′,4′,7-Tetrahydroxyflavone (Fisetin), 5-Hydroxyflavone, and 7-Hydroxyflavone and Antihemolysis Effects of Fisetin and Its Serum Metabolites. J. Agric. Food Chem..

[15] Huang M.-C., Hsueh T.Y., Cheng Y.-Y., Lin L.-C., Tsai T.-H. (2018). Pharmacokinetics and Biliary Excretion of Fisetin in Rats. J. Agric. Food Chem..

[16] Amidon G.L., Lennernäs H., Shah V.P., Crison J.R. (1995). A theoretical basis for a biopharmaceutic drug classification: The correlation of in vitro drug product dissolution and in vivo bioavailability. Pharm. Res..

[17] Ragelle H., Crauste-Manciet S., Seguin J., Brossard D., Scherman D., Arnaud P., Chabot G.G. (2012). Nanoemulsion formulation of fisetin improves bioavailability and antitumour activity in mice. Int. J. Pharm..

[18] Kulbacka J., Pucek A., Kotulska M., Dubińska-Magiera M., Rossowska J., Rols M.-P., Wilk K.A. (2016). Electroporation and lipid nanoparticles with cyanine IR-780 and flavonoids as efficient vectors to enhanced drug delivery in colon cancer. Bioelectrochemistry.

[19] Feng C., Yuan X., Chu K., Zhang H., Ji W., Rui M. (2019). Preparation and optimization of poly (lactic acid) nanoparticles loaded with fisetin to improve anti-cancer therapy. Int. J. Biol. Macromol..

[20] Danhier F., Ansorena E., Silva J.M., Coco R., Le Breton A., Préat V. (2012). PLGA-based nanoparticles: An overview of biomedical applications. J. Control. Release.

[21] Khalil N.M., Nascimento T.C.F.D., Casa D.M., Dalmolin L.F., Mattos A.C.D., Hoss I., Romano M.A., Mainardes R.M. (2013). Pharmacokinetics of curcumin-loaded PLGA and PLGA–PEG blend nanoparticles after oral administration in rats. Colloids Surf. B Biointerfaces.

[22] Wu Y.-T., Cham T.-M., Tsai T.-R. (2014). Development of HPLC with Photo-diode Array Method for the Determination of Ramipril in Tablets Using Factorial Design. J. Chin. Chem. Soc. TAIP.

[23] Paulo F., Santos L. (2017). Design of experiments for microencapsulation applications: A review. Mater. Sci. Eng. C..

[24] Song X., Zhao Y., Hou S., Xu F., Zhao R., He J., Cai Z., Li Y., Chen Q. (2008). Dual agents loaded PLGA nanoparticles: Systematic study of particle size and drug entrapment efficiency. Eur. J. Pharm. Biopharm..

[25] Feczkó T., Tóth J., Dósa G., Gyenis J. (2011). Optimization of protein encapsulation in PLGA nanoparticles. Chem. Eng. Process..

[26] Tefas L.R., Tomuţă I., Achim M., Vlase L. (2015). Development and optimization of quercetin-loaded PLGA nanoparticles by experimental design. Clujul. Med..

[27] Stolnik S., Garnett M.C., Davies M.C., Illum L., Bousta M., Vert M., Davis S.S. (1995). The colloidal properties of surfactant-free biodegradable nanospheres from poly(β-malic acid-co-benzyl malate)s and poly(lactic acid-co-glycolide). Colloids Surf. A. Physicochem. Eng. ASP.

[28] Sahoo S.K., Panyam J., Prabha S., Labhasetwar V. (2002). Residual polyvinyl alcohol associated with poly (d,l-lactide-co-glycolide) nanoparticles affects their physical properties and cellular uptake. J. Control. Release.

[29] Danaei M., Dehghankhold M., Ataei S., Hasanzadeh Davarani F., Javanmard R., Dokhani A., Khorasani S., Mozafari M.R. (2018). Impact of Particle Size and Polydispersity Index on the Clinical Applications of Lipidic Nanocarrier Systems. Pharmaceutics.

[30] Kadari A., Gudem S., Kulhari H., Bhandi M.M., Borkar R.M., Kolapalli V.R.M., Sistla R. (2017). Enhanced oral bioavailability and anticancer efficacy of fisetin by encapsulating as inclusion complex with HPβCD in polymeric nanoparticles. Drug Deliv..

[31] Ghosh P., Singha Roy A., Chaudhury S., Jana S.K., Chaudhury K., Dasgupta S. (2016). Preparation of albumin based nanoparticles for delivery of fisetin and evaluation of its cytotoxic activity. Int. J. Biol. Macromol..

[32] Sowa M., Ślepokura K., Matczak-Jon E. (2014). Improving solubility of fisetin by cocrystallization. CrystEngComm.

[33] Sechi M., Syed D.N., Pala N., Mariani A., Marceddu S., Brunetti A., Mukhtar H., Sanna V. (2016). Nanoencapsulation of dietary flavonoid fisetin: Formulation and in vitro antioxidant and α-glucosidase inhibition activities. Mater. Sci. Eng. C.

[34] Zhang J.-Q., Jiang K.-M., An K., Ren S.-H., Xie X.-G., Jin Y., Lin J. (2015). Novel water-soluble fisetin/cyclodextrins inclusion complexes: Preparation, characterization, molecular docking and bioavailability. Carbohydr. Res..

[35] Chen L.-F., Xu P.-Y., Fu C.-P., Kankala R.K., Chen A.-Z., Wang S.-B. (2020). Fabrication of Supercritical Antisolvent (SAS) Process-Assisted Fisetin-Encapsulated Poly (Vinyl Pyrrolidone) (PVP) Nanocomposites for Improved Anticancer Therapy. Nanomaterials.

[36] Zhang Z., Wang X., Zhu R., Wang Y., Li B., Ma Y., Yin Y. (2016). Synthesis and characterization of serial random and block-copolymers based on lactide and glycolide. Polym. Sci. Ser. B.

[37] Mistry P., Mohapatra S., Gopinath T., Vogt F.G., Suryanarayanan R. (2015). Role of the Strength of Drug–Polymer Interactions on the Molecular Mobility and Crystallization Inhibition in Ketoconazole Solid Dispersions. Mol. Pharm..

[38] SciFinder. https://scifinder.cas.org/scifinder/view/scifinder/scifinderExplore.jsf.

[39] Wang J., Zhao X.-H. (2016). Degradation kinetics of fisetin and quercetin in solutions as effected by pH, temperature and coexisted proteins. J. Serbian Chem. Soc..

[40] Krajčíková K., Suváková M., Glinská G., Ohlasová J., Tomečková V. (2020). Stability of natural polyphenol fisetin in eye drops Stability of fisetin in eye drops. Open Chem..

[41] Faisant N., Siepmann J., Benoit J.P. (2002). PLGA-based microparticles: Elucidation of mechanisms and a new, simple mathematical model quantifying drug release. Eur. J. Pharm. Sci..

[42] Song H.Y., Moon T.W., Choi S.J. (2019). Impact of antioxidant on the stability of β-carotene in model beverage emulsions: Role of emulsion interfacial membrane. Food Chem..

[43] Cao S.-J., Xu S., Wang H.-M., Ling Y., Dong J., Xia R.-D., Sun X.-H. (2019). Nanoparticles: Oral Delivery for Protein and Peptide Drugs. AAPS PharmSciTech.

[44] Zhao J., Yang J., Xie Y. (2019). Improvement strategies for the oral bioavailability of poorly water-soluble flavonoids: An overview. Int. J. Pharm..

[45] Ersoz M., Erdemir A., Derman S., Arasoglu T., Mansuroglu B. (2020). Quercetin-loaded nanoparticles enhance cytotoxicity and antioxidant activity on C6 glioma cells. Pharm. Dev. Technol..

[46] Luo H., Jiang B., Li B., Li Z., Jiang B.-H., Chen Y.C. (2012). Kaempferol nanoparticles achieve strong and selective inhibition of ovarian cancer cell viability. Int. J. Nanomed..

[47] Tariq M., Alam M.A., Singh A.T., Iqbal Z., Panda A.K., Talegaonkar S. (2015). Biodegradable polymeric nanoparticles for oral delivery of epirubicin: In vitro, ex vivo, and in vivo investigations. Colloids Surf. B Biointerfaces.

[48] Shailender J., Ravi P.R., Saha P., Dalvi A., Myneni S. (2017). Tenofovir disoproxil fumarate loaded PLGA nanoparticles for enhanced oral absorption: Effect of experimental variables and in vitro, ex vivo and in vivo evaluation. Colloids Surf. B Biointerfaces.

[49] Pawar A., Singh S., Rajalakshmi S., Shaikh K., Bothiraja C. (2018). Development of fisetin-loaded folate functionalized pluronic micelles for breast cancer targeting. Artif. Cells Nanomed. Biotechnol..

[50] Yen C.-C., Tung C.-W., Chang C.-W., Tsai C.-C., Hsu M.-C., Wu Y.-T. (2020). Potential Risk of Higenamine Misuse in Sports: Evaluation of Lotus Plumule Extract Products and a Human Study. Nutrients.

[51] Fatma S., Talegaonkar S., Iqbal Z., Panda A.K., Negi L.M., Goswami D.G., Tariq M. (2016). Novel flavonoid-based biodegradable nanoparticles for effective oral delivery of etoposide by P-glycoprotein modulation: An in vitro, ex vivo and in vivo investigations. Drug Deliv..

[52] Kumar S., Dilbaghi N., Saharan R., Bhanjana G. (2012). Nanotechnology as Emerging Tool for Enhancing Solubility of Poorly Water-Soluble Drugs. Bionanoscience.

[53] Yen C.-C., Chen Y.-C., Wu M.-T., Wang C.-C., Wu Y.-T. (2018). Nanoemulsion as a strategy for improving the oral bioavailability and anti-inflammatory activity of andrographolide. Int. J. Nanomed..

[54] Alam M.A., Al-Jenoobi F.I., Al-mohizea A.M. (2012). Everted gut sac model as a tool in pharmaceutical research: Limitations and applications. J. Pharm. Pharmacol..

