# Supplementary material for: Nanoformulation Development to Improve the Biopharmaceutical Properties of Fisetin Using Design of Experiment Approach
Source: Molecules. 2021 May 19;26(10):3031. doi: 10.3390/molecules26103031 (PMC8160650; doi:10.3390/molecules26103031)
Supplement: Supplementary file 1 [file molecules-26-03031-s001.zip › molecules-1222586-supplementary.pdf]

## Supplementary Materials

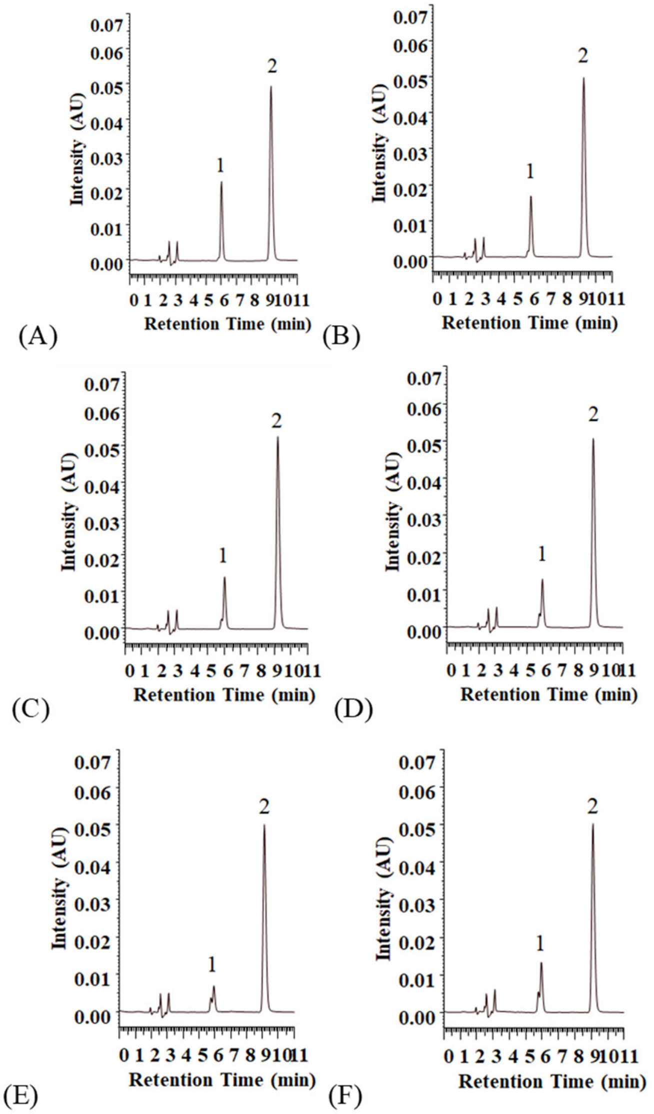


**Figure S1.** HPLC chromatography of FST-NP in pH 7.4 medium at different time points: (**A**) 8 h, (**B**) 24 h, (**C**) 48 h, (**D**) 72 h, (**E**) 96 h, (**F**) 120 h. 1: FST; 2: luteolin.
